# Facial Nerve Palsy with Total Ophthalmoplegia; a Novel Presentation of Fungal Invasion 

**DOI:** 10.22037/aaem.v9i1.1305

**Published:** 2021-07-28

**Authors:** Zainab Mehdi, Nidhi Bhardwaj, Jyoti Aggarwal, Narinder Kaur, Brijdeep Singh

**Affiliations:** 1 Department of General Medicine, Government Medical College and Hospital, Sector -32 Chandigarh, India.; 2 Department of Radio diagnosis, Government Medical College and Hospital, Sector -32 Chandigarh, India.; 3 Department of Pathology, Government Medical College and Hospital, Sector -32 Chandigarh, India.

**Keywords:** Mucormycosis, diabetes mellitus, facial paralysis, retinal artery occlusion

## Abstract

Mucormycosis is an expeditious invasion of a fungus of angioinvasive nature, predominant in immunocompromised individuals, often leading to organ malfunction and loss. Facial nerve involvement and total ophthalmoplegia are its rare presentations. Early detection and treatment can alter natural disease course and prevent potential catastrophic outcomes in diabetic patients. Facial nerve palsy is mostly attributed to peripheral neuropathy in patients with advanced diabetes mellitus. It rarely raises alarm about an invasive fungal infection. Here, we report the case of a 38-year-old male with type 2 diabetes mellitus, who presented to us with left lower motor neuron type facial palsy and left-sided total ophthalmoplegia due to invasive rhino-orbito-cerebral mucormycosis (ROCM). Despite aggressive measures, including antifungal therapy and repeated endoscopic debridement, he subsequently developed central retinal artery occlusion (CRAO) and underwent left eye exenteration.

## 1. Introduction:

Mucormycosis, coined and reclassified by R.D.Baker, is an insidious fungal infection caused by ubiquitous mold, mucormycetes, which belongs to family of mucoraceae, order Mucorales, class zygomycetes (1, 2).

Since being reported first in 1885 by German pathologist Paltauf, they have increasingly manifested themselves primarily among immunocompromised individuals (1, 2). A 2009 French study reviewing 10-year trends of mucormycosis reported 7.4% annual amplification of such cases (3). Diabetes mellitus is the most common predisposing factor, the rest include hematological neoplastic diseases, iron overload, and steroid and deferoxamine therapy (4, 5).

The clinical presentation has been broadly divided into five main categories: rhino-orbital-cerebral, pulmonary, cutaneous, gastrointestinal, and disseminated. Rhino-orbital-cerebral mucormycosis (ROCM) can be in acute or the lesser-known chronic form. Acute form spreads aggressively involving nose, sinus, orbit, and other cranial entities within a short period of time, typically manifesting as orbital swelling, headache, ophthalmoplegia, or visual loss (6). Facial nerve palsy is a relatively lesser-known and unusual presentation of ROCM. It has the potential of being misdiagnosed as a vascular event, causing delayed detection and treatment, while adversely affecting the outcome (7). Similarly, central retinal artery occlusion (CRAO), lower cranial nerves palsy (IX, X CN), and cavernous sinus thrombosis have also been documented as rare presenting symptoms of unilateral ROCM (8, 9).

Here, we report a case of a 38-year-old male with type II diabetes mellitus who had advanced intracranial and intraorbital mucormycosis. The presence of unilateral lower motor neuron (LMN) type facial palsy and ipsilateral total ophthalmoplegia as presenting features of fungal infection make this case unique.

## 2. Case presentation:

A 38-year-old male presented to the emergency medicine department of our urban academic tertiary care center with painless left eye swelling for the last seven days. He also complained of progressive limitation of left eye movement with drooping of left eyelid, ultimately causing inability to open left eye over last week. He complained of having double vision while looking sideways. While looking himself in mirror two days ago, he also noticed his mouth deviating towards the right side of his body. He had history of high-grade fever, mostly at nighttime, in the last fifteen days, which was responsive to oral antipyretics, as well as few episodes of bloody sputum. His wife also noticed his body excessively sweating at night for the last few weeks and his significant weight loss in the last few months.

The patient had suffered from diabetes mellitus for the last seven years, non-compliant to orally administered anti-hyperglycemic agents (OHA) treatment. His past medical history revealed having been diagnosed with pulmonary tuberculosis two years back, for which he took regular treatment only for four months. He has smoked (30 pack years), consumes alcoholic beverages four days a week, and occasionally uses opium. There was no history of trauma, involuntary body movements, pus or watery discharge from ear, epistaxis, alteration in sensorium or behavior, dysphagia, or change in voice. No history of any recent surgery was present. 

**Figure 1:**
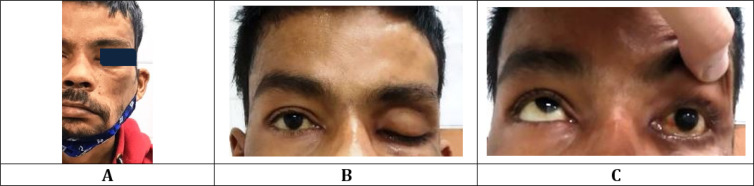
(A) left facial droop and deviation of mouth to right side; (B) left sided absence of forehead wrinkling and left sided ptosis; (C) inability to open eye and absent eyeball movement on left side upon being asked to look up

The patient was conscious with Glasgow coma score (GCS) of E3V4M5, afebrile (temp 99.6 F), blood pressure of 100/80 mmHg, pulse rate of 89/min, and respiratory rate of 22/min, cooperative with physician. Neurological examination revealed deviation of angle of mouth to the right side with absent wrinkling of forehead on left side, abnormal grimace with hypoesthesia in distribution of V1, V2 branches of left trigeminal nerve (Figure 1). On blowing of mouth air, leak was present from left side. Left eye ptosis, immobility, fixed mid-dilated non-reactive pupil and complete loss of accommodation, and loss of left corneal sensation were noted. Fundoscopy revealed normal disc and macula. He had bilateral lower and upper limb power of 5/5 with normal reflexes and no other cranial nerve deficit was noted. Nasal discharge from left nostril was seen, which was not blood stained. No infraorbital necrosis, ulcer, or perforation of hard palate were present. Gag reflex was intact.

**Figure 2:**
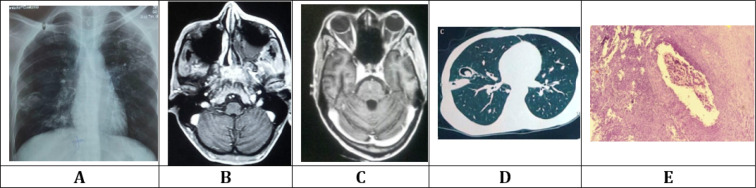
(A) Chest X-ray posteroanterior (PA) view: There are right upper and lower zone cavitary lesions showing radiopacity in dependant parts and air-crescent sign along the non-dependant parts. Multiple alveolar infiltrates and llinear strands are seen in left upper and middle zones; (B) Contrast-enhanced magnetic resonance imaging (CEMRI) of brain; T_1 _(axial scan at the level of base of skull) showing enhancenment of basisphenoid, clivus, and basiocciput. Contrast enhancement is extending to left sided retroantral fat and posterolateral wall of left maxillary sinus and contents of left infratemporal fossa. Left maxillary sinus shows mucosal thickening along the posteromedial wall, air fluid level and some hyperdensity along the medial wall. All these findings are suggestive of invasive fungal sinusitis; (C) CEMRI of brain (axial scan) at the level of cavernous sinus and orbits showing post contrast enhancement of left optic canal and optic nerve. A small filling defect is noted in left cavernous sinus as well as outward bulge of the lateral wall, which indicates thrombosis; (D) Consultation contrast-enhanced CT scan (CECT) of chest showing cavitary lesion in right middle lobe with air crescent sign along the non-dependant part and ball-like soft tissue along the dependant part of cavity. Adjacent lung parenchyma posterolaterally shows consolidaion and thickening of abutting right oblique fissure; (E) Haematoxylin and eosin (H&E) stain (20x10) fungal profiles can be seen invading vessel wall accompained by dense inflammatory cell infiltrate comprising neutrophils, lymphocyte, and few scattered eosinophils and plasma cells

Based on clinical findings, after ruling out possibility of trauma, a differential diagnosis of cerebrovascular accident (CVA), intracranial space occupying lesion, tuberculoma or diabetes mellitus-related neuropathy with tuberculosis reactivation were considered. Initial Non-Contrast computed tomography (CT) scan (NCCT) of the head revealed left maxillary sinusitis. Hematological and biochemical workup done in emergency department revealed anemia, raised total leukocyte count (TLC), and high random blood glucose without ketoacidosis (Table 1). Cerebrospinal fluid examination was unremarkable. Electrocardiogram (ECG) was not suggestive of any abnormality. Urine and blood culture were sterile. Sample for assessing KOH mount of nasal secretion was sent. His sputum was sent for acid fast bacilli (AFB) staining as well as fungal evaluation. Patient was admitted to the emergency medicine ward and started on intravenous (IV) antibiotics empirically, and insulin for glycemic control, as further diagnostic evaluations continued. As per local guideline, his high nasal and oropharyngeal swab were sent for RTPCR for COVID-19, which came negative. His chest radiograph revealed fibrotic bands along with cavitary lesion with surrounding consolidation and air crescent sign present in both hemi thoraces (figure 2).

Contrast-enhanced magnetic resonance imaging (CEMRI) of brain, orbit, and paranasal sinuses (PNS) was done to look for intracranial pathology. It demonstrated acute left maxillary sinusitis with posterolateral wall erosion, prominent left optic nerve sheath, and skull base osteomyelitis with cavernous sinus thrombosis (Figure 2). In view of chest X-ray findings, after pulmonary consultation contrast-enhanced CT scan (CECT) of chest was performed to aid in decision regarding initiation of anti-tubercular therapy, which suggested tuberculosis reactivation (Figure 2). Patient’s KOH mount of nasal secretion showed non-septate ribbon-like hyphae.

Based on cumulative results of initial evaluation, a primary diagnosis of invasive rhino-orbito-cerebral mucormycosis with pulmonary tuberculosis reactivation was made. Anti-tubercular treatment (ATT) was started and ear, nose, throat (ENT) review was sought for assessing need for urgent endoscopic debridement, taking in account intra-orbital and intracranial fungal extension causing multiple cranial nerve palsies threatening vision. Nasal endoscopic debridement and left orbital decompression were performed the next day. Histopathological examination (HPE) of excised tissue revealed broad aseptate hyphae with right angle branching invading both nerves and vessel wall, confirming diagnosis of invasive mucormycosis (Figure 2).

Patient was started on amphotericin B, optimal anticoagulation, and insulin therapy for optimization of glycemic control. His biochemistry was regularly monitored for any nephrotoxicity, hematotoxicity, and other infusion-related adverse effects. A repeat endoscopic debridement was performed after a period of seven days. Meanwhile, serial fundoscopy was performed by ophthalmologist to look for possible optic nerve involvement, any optic disc changes, papilledema, or decrease in visual acuity and field. A week after the second debridement and thirteen days of amphotericin B cumulative dose 1425 milligrams, patient complained of severe pain in left side of face, predominant in the orbital region with increased swelling on waking up from sleep. Fundus examination revealed a cherry red spot, optic disc edema with complete retinal opacification, suggesting central retinal artery occlusion in left eye. Patient was started on topical timolol 0.5% and systemic acetazolamide alongside intermittent digital ocular massage with the aim of reducing intraocular pressure. He did not respond to the above measures and decision for left eye exenteration alongside repeat decompression and debridement was made. Post-exenteration monitoring for involvement of right eye and other signs of raised intraocular and intracranial pressures continued alongside antifungal therapy. His condition remained satisfactory and gradual improvement followed. His right eye vision remained unaltered and serial fundoscopy did not reveal any alarming features. Optimal glycemic control was achieved with insulin therapy. He was ultimately discharged home after 48 days of hospital stay in a stable state with residual left facial nerve palsy, written advice to follow up for dressing, ocular prosthesis, and further treatment optimization.

## 3. Discussion:

Mucormycosis, a deadly fungal infection by Mucorales, nearly invariably involving immunocompromised patients, especially diabetics, can disseminate to paranasal sinuses, retro orbital regions, and brain. Its ability to extensively and uniformly invade vessels facilitates hematogenous spread to various areas leading to vessel thrombosis and tissue necrosis in multiple organs (10).

Factors that render diabetics more prone to fungal invasion include pre-existing nerve ischemia and injury, abnormal endoneurial and epineural vessels, resistant arteries, reduced chemotaxis and phagocytic efficiency, and favorable acidic and high-sugar environment helping hyphae production (7, 11). Pterygopalatine fossa plays a key role in involvement of facial nerve and retro global area. Its numerous vascular and neural connections facilitate cranial invasion and lower cranial nerve palsies (7). Our patient had fungal skull base osteomyelitis, which may have facilitated facial nerve invasion via pterygopalatine fossa, cavernous sinus thrombosis causing ipsilateral involvement of third, fourth and sixth cranial nerves manifesting as total ophthalmoplegia and sensory loss in distribution of V1 and V3 divisions of the fifth cranial nerve.

Prognosis of mucormycosis with intracranial and intra-orbital extension is disastrous and usually fatal. Amalgamation of surgical debridement and amphotericin B has improved patient survival to 85% from 24% in those untreated (12). Our patient was managed with early and multiple-staged endoscopic debridement. He received a total dose of 4125 milligrams of amphotericin B. Due to advanced fungal invasion of left optic nerve and subsequent thrombosis of ophthalmic artery, his left eye could not be salvaged. 

Knowing that unilateral facial nerve palsy can be a presenting feature of intracranial spread of fungal infection and considering it in the initial workup of all immunocompromised patients presenting with peripheral neuropathy can help early cessation of disease advancement and salvage of organs. Multiple cranial nerve palsies in an immunocompromised patient without history of trauma should alarm emergency physician of possible intracranial fungal infection. It is of outmost importance to assess KOH mount of nasal secretions, keep a low threshold for CEMRI of brain, orbit, and PNS and start antifungal treatment as early as possible. Long duration of amphotericin therapy demands close monitoring and intervention for any drug side effects. Continual monitoring for any new sign and symptom is recommended as patients tend to develop other complications while on treatment, like CRAO in our case.

**Table 1:** Results of laboratory investigations performed during the hospital stay

**Variables**	**Day1**	**Day7**	**Day14**	**Day21**	**Day28**	**Day32**	**Day40**
Hemoglobin (g/dl)	10.7	9.8	10.2	11.7	12	11.6	12.1
Leukocyte count(10^9^cells/L)	12.3	13.3	16.9	11.1	9.8	6.1	6.3
Polymorphs (%)	79.1	60	66	67	70	71	68
Lymphocytes (%)	8.7	8.5	8.2	7.3	8.1	6.8	7.1
Eosinophils (%)	3.2	3.2	3.1	2.9	2.9	3	3.3
Basophils (%)	1.6	1.4	1.2	1.6	1.1	1.5	1.4
Monocytes (%)	0.4	0.6	0.6	0.3	0.8	0.6	0.4
Platelet count (lac/mm^3^)	475	471	506	696	423	413	415
ESR* (mm/hour)	12		10		10		8
Total serum bilirubin (mg/dL)	0.1			1.3			0.7
Conjugated bilirubin (mg/dL)	.3			.4			.2
Alkaline phosphatase (U/L)	70	69	70	101	72	68	66
AST (U/L)	18	40	130	135	118	98	45
ALT (U/L)	27	47	143	147	125	103	53
Total protein	6.4		7.6			5.7	5.9
Albumin	3.5		2.8			3.1	3.0
Serum sodium (mEq/L)	136	137	125	129	136	139	141
Serum potassium (mEq/L)	4.7	4.1	3.6	3.8	4.1	4.5	4.4
Chloride (mEq/L)							
Blood urea (mg/dL)	21	23	37	32	28	20	16
Serum creatinine (mg/dL)	0.6	0.7	0.9	0.5	0.6	0.6	0.7
Calcium (mg/dL)	9.1	8.9	8.2	8.3	8.7	9.1	9.3
Magnesium (mg/dL)	2.7	2.2	1.9	1.8	1.9	2.7	3
Phosphorus (mg/dL)	2.9	3.8	2.9	2.6	2.7	2.8	2.9
FBS (mg/dL)	286	228	237	195	121	201	198
pH*	7.34	7.34	7.23	7.24	7.34	7.35	7.41
PaO_2_	88	89	88	87	88	88	88
PaCO_2_	44	36	38	42	43	42	45
HCO_3_	23	28	26	24	24	22	24
SpO_2 _(%)	89	94	93	95	96	94	93
Lactate	1.4	1.6	1.7	.9	.9	1.0	.9
Urine ketone	Nil				Nil		
HbA_1_C	13						

*: Arterial blood gas analysis. AST: aspartate aminotransferase, ALT: alanine transaminase, LDH: lactate dehydrogenase, SpO2: peripheral oxygen saturation, ESR: erythrocyte sedimentation rate, FBS: fasting blood sugar.

## 4. Conclusion:

Mucormycosis infection in immunocompromised individuals can diverge considerably: from being a simple acute fungal sinusitis to deadly orbital and cranial extension. Therefore, an approach based on early suspicion, detection, and intervention, resulting in favorable outcome and preventing complications, is commendable. 

## 5. Declaration:

### 5.1 Acknowledgment

None

### 5.2 Authors' contribution

All the authors have made substantial contributions to conception and design, or acquisition of data, or analysis and interpretation of data. All the authors have been involved in drafting the manuscript or revising it critically for important intellectual content and have given final approval of the version to be published. Each author has participated sufficiently in the work to take public responsibility for appropriate portions of the content. The corresponding author takes responsibility for the article during the submission and review process.

Dr Zainab Mehdi, Concept, design, intellectual content, literature search, data acquisition, manuscript preparation, editing, and review

Dr Nidhi Bhardwaj, Concept, design, intellectual content, literature search, editing, and review

Dr Jyoti Aggarwal, Literature search, manuscript preparation, data acquisition, manuscript editing and review

Dr. Narinder Kaur, Literature search, clinical studies, data acquisition, manuscript preparation, editing, and review

Dr. Brijdeep Singh, Literature search, clinical studies, data acquisition, manuscript preparation, editing, and review

### 5.3 Funding and support

None

### 5.4 Conflict of interest

None declared

### 5.5 Ethical considerations and patient's consent

The authors certify that they have obtained all appropriate patient consent forms. In the form, the patient has given his consent for his images and other clinical information to be reported in the journal. The patient understands that name and initials will not be published and due efforts will be made to conceal the identity, but anonymity cannot be guaranteed.
